# Association of *CASR, CALCR*, and *ORAI1* Genes Polymorphisms With the Calcium Urolithiasis Development in Russian Population

**DOI:** 10.3389/fgene.2021.621049

**Published:** 2021-05-12

**Authors:** Maria M. Litvinova, Kamil Khafizov, Vitaly I. Korchagin, Anna S. Speranskaya, Aliy Yu. Asanov, Alina D. Matsvay, Daniil A. Kiselev, Diana V. Svetlichnaya, Sevda Z. Nuralieva, Alexey A. Moskalev, Tamara V. Filippova

**Affiliations:** ^1^Department of Medical Genetics, Ministry of Public Health of the Russian Federation, I. M. Sechenov First Moscow State Medical University, Sechenov University, Moscow, Russia; ^2^Moscow Health Department, The Loginov Moscow Clinical Scientific Center, Moscow, Russia; ^3^Moscow Institute of Physics and Technology, National Research University, Dolgoprudny, Russia; ^4^Federal Service on Consumers’ Rights Protection and Human Well-Being Surveillance, Central Research Institute for Epidemiology, Moscow, Russia; ^5^Center of Strategic Planning of FMBA of Russia, Moscow, Russia; ^6^Moscow Regional Research and Clinical Institute (MONIKI), Moscow, Russia; ^7^Center for Precision Genome Editing and Genetic Technologies for Biomedicine, Engelhardt Institute of Molecular Biology, Russian Academy of Sciences, Moscow, Russia

**Keywords:** kidney stone disease, urolithiasis, calcium stones, calcium urolithiasis, *CALCR*, *CASR*, *ORAI1*, *CLDN14* gene

## Abstract

Kidney stone disease is an urgent medical and social problem. Genetic factors play an important role in the disease development. This study aims to establish an association between polymorphisms in genes coding for proteins involved in calcium metabolism and the development of calcium urolithiasis in Russian population. In this case-control study, we investigated 50 patients with calcium urolithiasis (experimental group) and 50 persons lacking signs of kidney stone disease (control group). For molecular genetic analysis we used a previously developed gene panel consisting of 33 polymorphisms in 15 genes involved in calcium metabolism: *VDR*, *CASR*, *CALCR*, *OPN*, *MGP*, *PLAU*, *AQP1*, *DGKH*, *SLC34A1*, *CLDN14*, *TRPV6*, *KLOTHO*, *ORAI1*, *ALPL*, and *RGS14.* High-throughput target sequencing was utilized to study the loci of interest. Odds ratios and 95% confidence intervals were used to estimate the association between each SNP and risk of urolithiasis development. Multifactor dimensionality reduction analysis was also carried out to analyze the gene-gene interaction. We found statistically significant (unadjusted *p*-value < 0.05) associations between calcium urolithiasis and the polymorphisms in the following genes: *CASR* rs1042636 (OR = 3.18 for allele A), *CALCR* rs1801197 (OR = 6.84 for allele A), and *ORAI1* rs6486795 (OR = 2.25 for allele C). The maximum OR was shown for AA genotypes in loci rs1042636 (*CASR*) and rs1801197 (*CALCR*) (OR = 4.71, OR = 11.8, respectively). After adjustment by Benjamini-Hochberg FDR we found only *CALCR* (rs1801197) was significantly associated with the risk of calcium urolithiasis development. There was no relationship between recurrent course of the disease and family history of urolithiasis in investigated patients. Thus we found a statistically significant association of polymorphism rs1801197 (gene *CALCR*) with calcium urolithiasis in Russian population.

## Introduction

Kidney stone disease (KSD) has been known to be one of the most excruciating chronic diseases. It is estimated to affect nearly 5% of women and 12% of men during their lifetime, and is considered to be the third most frequent urological disorder (Worcester and Coe, 2010). Multiple studies have revealed that genetics alter the risk of KSD development alongside the environmental factors. It is believed that the vast majority of cases are, in fact, multifactorial.

Deciphering the molecular substrate for the etiopathogenesis of urolithiasis is of outmost importance for developing diagnostic tools and therapy strategies. In most cases, KSD is caused by the formation of calcium concrements, supplying grounds for research into the calcium metabolism impairments in those affected by the disease. Numerous works have been published that elucidate hidden associations between polymorphisms in genes of calcium metabolism and the development of KSD (Filippova et al., 2020). To our knowledge, very few investigations were performed to look into these associations in Russian population (Apolihin et al., 2015; Apolikhin et al., 2016, 2017). According to various reports, the development of calcium urolithiasis has been attributed to polymorphisms in several genes: *VDR, CASR, CALCR, OPN, MGP, PLAU, AQP1, SLC34A1, CLDN14, KLOTHO*, and *ORAI1* (Filippova et al., 2020).

In this article, we examine possible connections between polymorphisms in genes of calcium metabolism and the risk of KSD development in Russian population.

## Materials and Methods

### Patients Characteristics

In this case-control study, the experimental group featured 50 patients with KSD, and the control group consisted of 50 healthy individuals aged 1 to 70. All patients suffered from calcium oxalate urolithiasis (as verified with spectral assay of the concrements). The distribution of patients by gender in both groups is following: 13 male patients (26%), 37 female patients (74%). The mean age at onset of the disease in the group of patients with urolithiasis was 29.6 years (median 24 years). The average age of the subjects at the time of participation in the study in both groups was 38.5 years (median – 34 years). Family history on KSD was collected from all patients. No family history of KSD was found for any people in the control group.

The study was confirmed by the ethics committee of Sechenov University. All participants signed informed consent prior to entering the research program.

### Genetic Analysis

A previously developed gene panel was used to evaluate possible correlations between the development of KSD and polymorphisms in the genes of calcium metabolism: *VDR* (rs1544410, rs731236), *CASR* (rs6776158, rs7652589, rs1501899, rs1801725, rs1042636, rs1801726), *CALCR* (rs1042138, rs1801197), *OPN* (rs2853749, rs2853750, rs1126616, rs4754), *MGP* (rs4236), *PLAU* (rs4065), *AQP1* (rs12669187, rs1000597), *DGKH* (rs4142110), *SLC34A1* (rs12654812), *CLDN14* (rs219781, rs219780, rs219779, rs219778, rs219777), *TRPV6* (rs4987667, rs4987682), *KLOTHO* (rs3752472), *ORAI1* (rs12313273, rs6486795, rs7135617), *ALPL* (rs1256328), and *RGS14* (rs11746443) (Filippova et al., 2020).

Peripheral blood was used as a source of genomic DNA. The DNA was extracted with DNeasy Blood & Tissue Kit (Qiagen) on QIAcube automated extraction platform (Qiagen) according to the manufacturer’s instructions.

Obtained DNA was PCR-amplified with a primer panel specifically developed for this study. The panel featured 68 primers divided into 2 pools to optimize amplification and minimize possible artifacts. Target PCR was conducted with AmpliSens reagents on QuantStudio 5 real-time PCR system (Thermo Fisher Scientific).

NGS libraries were prepared according to an in-house protocol. T4 Polynucleotide Kinase and T4 DNA Ligase (both New England Biolabs) were utilized in compliance with the manufacturer’s directions with slight modifications to maximize the output.

PCR products and NGS libraries at any stage of library preparation were purified with Sera-Mag SpeedBeads (General Electric) according to the manufacturer’s protocol to ensure recovery of the fragments of an optimal length. DNA concentrations were measured with Qubit 2.0 Fluorometer (Thermo Fisher Scientific) using Qubit^TM^ dsDNA High Sensitivity Assay Kit (Thermo Fisher Scientific). The quality of the final libraries was assessed with on-chip capillary electrophoresis on Agilent 2100 Bioanalyzer (Agilent Technologies) with Agilent High Sensitivity DNA Kit (Agilent Technologies).

The libraries were sequenced on Ion S5 (Thermo Fisher Scientific) high-throughput sequencing platform with Ion 520 & Ion 530 Kit-Chef (Thermo Fisher Scientific) reagents on Ion 530 Chip (Thermo Fisher Scientific).

### Bioinformatic Analysis of Sequencing Data

The analysis of primary sequencing data included several stages: (1) read quality filtering with PRINSEQ-lite (Schmieder and Edwards, 2011); (2) mapping to the reference human genome (GRCh38.p7, PRJNA31257) with Burrows-Wheeler Aligner (BWA-mem, v_0.7.13) (Schmieder and Edwards, 2011); (3) searching for single-nucleotide variants (SNVs) with Genome Analysis Toolkit (GATK version: 4.0.11.0) (McKenna et al., 2010). SAMtools v_1.3.1 (Li et al., 2009) and Picard toolkit v_2.18.17 were used for working with sam/bam files. VEP tool (McLaren et al., 2016) combined with 94_GRCh38 cash was used for primary variant annotation. Identified SNVs were validated manually in Tablet (Milne et al., 2013).

### Statistical Analysis

The final matrix of 28 SNPs obtained after removing SNPs with high linkage disequilibrium, was using for association studies by PLINK v1.90b6.9 (Steiß et al., 2012; Chang et al., 2015). Statistical analyses were conducted with the standard functions of the R environment and packages (R Core Team, 2019). Differences in allelic and genotypic distributions were estimated by Fisher‘s exact test with Lancaster’s mid-p adjustment (Lancaster, 1961). Hardy-Weinberg equilibrium (HWE) in controls was calculated using the chi-squared test with continuity correction (Graffelman, 2015). To estimate the association between each SNP and KSD risk the odds ratios (OR) and 95% confidence intervals (CIs) were calculated using exact methods (median-unbiased estimation (mid-p), maximum likelihood estimation (Fisher) and small sample adjustment) by Epitools package (Tomas, 2017). Differences with the *p*-value less than 0.05 were considered statistically significant.

For detecting multilocus genotype combinations which may predict disease risk, multifactor dimensionality reduction (MDR) approach was used by MDR 3.0.2 (build 2) software package (Hahn et al., 2003). MDR is a non-parametric data mining method that assumes no genetic model and has been supported by numerous studies of gene-gene and gene-environment interactions (Ritchie et al., 2001; Cho et al., 2004; Andrew et al., 2006; Brassat et al., 2006). Cross-validation and 1000-fold permutation testing were used to find optimal models for defining disease risk.

## Results

Data analysis revealed a statistically significant association between the development of calcium KSD and polymorphisms in the following genes: *CASR* (rs1042636, OR = 3.18), *CALCR* (rs1801197, OR = 6.84), *ORAI1* (rs6486795, OR = 2.25). Association between SNP rs219780 of the *CLDN14* gene and urolithiasis was characterized by borderline *p*-value (OR = 2.03; *p* = 0.05). After the adjustment by Benjamini-Hochberg procedure we found only *CALCR* (rs1801197) significantly associate with the risk of calcium urolithiasis development (Table 1). For other studied loci of the gene panel, no statistically significant differences in allele frequencies were found between the experimental and control groups.

**TABLE 1:** Associations between the risk of calcium urolithiasis development and polymorphisms of *CASR, CALCR, ORAI1*, and *CLDN14* genes.

Gene	SNP, (risk allele)	Allele frequency		OR (95% CI)	*p*-value*	Permutation p-value**	FDR BH***
		Case, %	Control, %				
*ORAI1*	rs6486795 (C)	30%	16%	2.25 (1.135–4.462)	0.020	0.036	0.33
*CALCR*	rs1801197 (A)	94%	69%	6.84 (2.87–19.26)	0.000004 (<0.0001)	0.00002	0.00012
*CASR*	rs1042636 (A)	96%	88%	3.18 (1.05–12.07)	0.041	0.046	0.43
*CLDN14*	rs219780 (C)	86%	75%	2.03 (0.99–4.31)	0.052	0.059	0.43

*Fisher’s exact test with Lancaster’s mid-p adjustment was used to obtain *p*-values. **Adaptive Monte Carlo permutation test was performed. ***Benjamini-Hochberg FDR adjustment for 28 SNP.

More than a half of the patients from the experimental group (26 patients, 52%) had a family history of KSD. In the group of patients with KSD 26 persons (52%) suffered from recurring urolithiasis. Among patients with recurring urolithiasis, 14 people (53.9%) had a family history of KSD. Among those with non-recurring urolithiasis 12 patients (50%) had a family history of the disease. Thus, no relationship was found between the recurrent course of the disease and the family history of the patients (Pearson’s Chi-squared test with Yates’ continuity correction, χ^2^ = 0, *p-*value = 1).

Comparative characteristics of genotype frequencies of genes loci *CASR* (rs1042636), *CALCR* (rs1801197), *ORAI1* (rs6486795), and *CLDN14* (rs219780), affecting the risk of KSD in our study, is shown in Table 2. Genotype distributions for all loci were compatible with HWE in controls. For the loci of the *CASR* and *CALCR* genes, a statistically significant difference was shown between the experimental and control groups, both in the frequency of the alleles and in the frequency of genotypes (*p* < 0.05).

**TABLE 2:** Genotypes frequencies of loci associated with KSD development in the group of patients with calcium urolithiasis and in the control group.

Locus	Genotype	Genotype frequency		Fisher’s exact test, *p-value*/adjusted p-value*	HWE in controls
		Case, n (%)	Control, n (%)		
rs1042636 *(CASR)*	AA	47 (94)	38 (76)	0.008/0.016	0.70
	AG	2 (4)	12 (24)		
	GG	1 (2)	0		
rs1801197 *(CALCR)*	AA	46 (92)	24 (48)	0.0000011/0.000016	0.88
	AG	2 (4)	21 (42)		
	GG	2 (4)	5 (10)		
rs6486795 *(ORAI1)*	TT	25 (50)	36 (72)	0.074/0.52	0.74
	TC	20 (40)	12 (24)		
	CC	5 (10)	2 (4)		
rs219780 (*CLDN14)*	CC	37 (74)	28 (56)	0.212/0.52	0.82
	CT	12 (24)	19 (38)		
	TT	1 (2)	3 (6)		

*Benjamini-Hochberg FDR

Table 3 shows the significance of the dominant and recessive models for the studied polymorphisms of the *CASR* (rs1042636), *CALCR* (rs1801197), *ORAI1* (rs6486795), and *CLDN14* (rs219780) genes regarding the development of calcium urolithiasis in the Russian population.

**TABLE 3:** Association of the *CASR*, *CALCR*, *ORAI1*, and *CLDN14* genes genotypes with the risk of calcium urolithiasis development under the different inheriting models.

Locus	Model	Group		OR (95% CI)	*p*-value*
		Case	Control		
rs1042636 *(CASR)*	Dominant (AA + AG vs. GG)	49/1	50/0	0 (0.01–8.2)***	0.5
	Recessive (AA vs. AG + GG)	47/3	38/12	4.71 (1.36–23.0)	0.013/0.052**
rs1801197 *(CALCR)*	Dominant (AA + AG vs. GG)	48/2	45/5	2.54 (0.49–20.5)	0.27
	Recessive (AA vs AG + GG)	46/4	24/26	11.8 (4.0–44.9)	0.000001/0.000008**
rs6486795 *(ORAI1)*	Dominant (CC + CT vs. TT)	25/25	14/36	2.54 (1.11–5.97)	0.027/0.072**
	Recessive (CC vs. CT + TT)	5/45	2/48	2.53 (0.49–20.5)	0.274
rs219780 (*CLDN14)*	Dominant (CC + CT vs. TT)	49/1	47/3	2.85 (0.32–83.9)	0.367
	Recessive (CC vs. CT + TT)	37/13	28/22	2.21 (0.96–5.28)	0.064

*Fisher’s exact test with Lancaster’s mid-p adjustment was used to obtain *p*-values. **Benjamini-Hochberg FDR ***OR was calculated by small sample adjustment.

Under the recessive model of inheritance, carriers with the AA genotypes of *CASR* (rs1042636) and AA genotypes of *CALCR* (rs1801197) had a 4.71-fold and 11.8-fold increased risk of KSD respectively. Moreover the carriers of CC/CT genotype of rs6486795 *(ORAI1)* have 2.54-fold increased risk of KSD comparing to carriers of TT genotype. After the adjustment by Benjamini-Hochberg procedure no statistically significant differences between KSD patients and controls were found for the rs219780 (*CLDN14*) and rs1042636 *(CASR)*.

**FIGURE 1:**
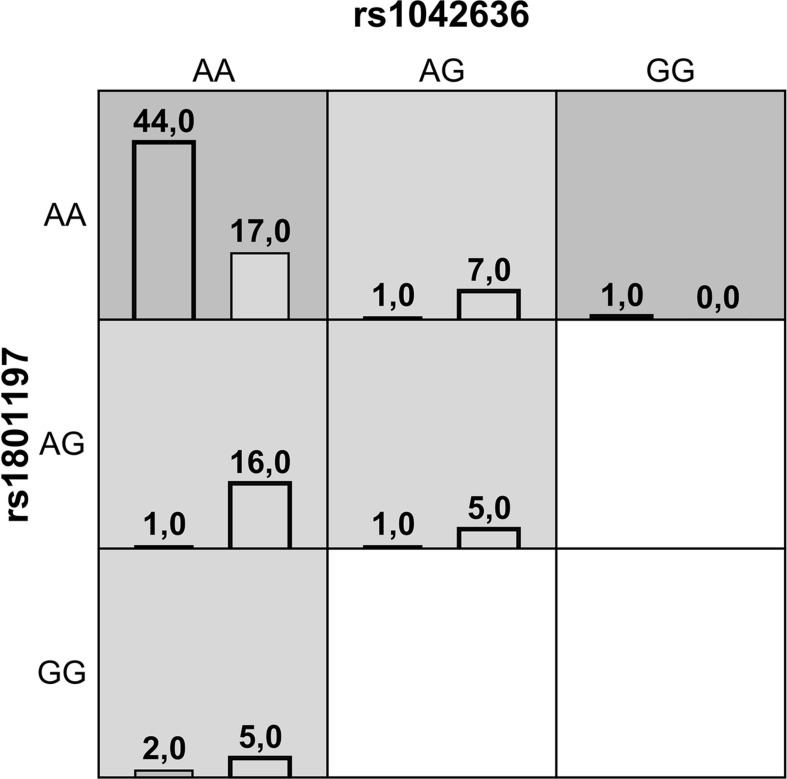
A summary of the best two-way gene-gene interaction analysis by multifactor dimensionality reduction for 9 genotypes [rs1042636 (*CASR*) and rs1801197 (*CALCR*)] associated with increased risk of KSD. The dark shading box represents high-risk combinations and the light shading box shows low-risk combinations. The left and right columns represent the absolute number of the cases and controls, respectively.

Gene-gene interaction analysis using MDR approach showed that a two-locus model consisting of rs1042636 *(CASR)* and rs1801197 *(CALCR)* might have a non-linear association with the susceptibility to the KSD development. This model had an overall accuracy test of 78%, a consistency of cross-validation of 9/10, and a 1000-fold permutation *p*-value = 0.003 (Table 4). Figure 1 summarizes the two-way gene interaction showing the high-risk genotype [AA + AA] of the rs1042636 (*CASR*) and rs1801197 (*CALCR*) associated with an increased KSD risk (OR = 2.59, 95% CI = 1.78-3.86).

**TABLE 4:** Interaction analysis of SNPs in *CASR* and *CALCR* and risk of KSD development.

Best candidate models	Training Bal. Acc. (%)	Testing Bal. Acc. (%)	Overall Bal. Acc. (%)	CV consistency	*p*-value
rs1801197	72	72	72	10/10	0.007
rs1042636/rs1801197	78	74	78	9/10	0.003

*CV, cross-validation; Bal, balanced; Acc, accuracy.*

## Discussion

We detected an association between the polymorphisms of *CASR* (rs1042636), *CALCR* (rs1801197), and *ORAI1* (rs6486795) genes and the development of calcium urolithiasis in the Russian population. However after adjustment by Benjamini-Hochberg FDR we found only *CALCR* (rs1801197) was significantly associated with the risk of calcium urolithiasis development.

It is known that these genes products are involved in calcium metabolism. Thus the *CASR* gene encodes a calcium-sensing receptor which senses changes of calcium concentration in an organism and controls a parathyroid hormone secretion. Activation of the parathyroid hormone synthesis stimulates the calcium release from bone tissue into the bloodstream and decreases the phosphates and calcium reabsorption in the proximal renal tubules (Vezzoli et al., 2013). The *CALCR* gene is attributable for a calcitonin receptor synthesis. CALCR interacts with the hormone calcitonin which is a functional antagonist of a parathyroid hormone and inhibits the activity of osteoclasts in the bone tissue. This in turn decreases calcium release into the bloodstream and also regulates the phosphates and calcium reabsorption in the renal tubules (Shakhssalim et al., 2014). The *ORAI1* gene encodes calcium release–activated calcium modulator type 1. This protein is required for transmembrane calcium metabolism. It is usually activated upon the depletion of internal calcium stores (Chou et al., 2011).

The association between the *CASR*, *CALCR*, and *ORAI1* genes polymorphisms and the urolithiasis development has been shown in a number of studies conducted in Italian, Indian, and Iranian, Chinece populations by different researchers. The data obtained in this study are generally consistent with the data of the world literature (Corbetta et al., 2006; Scillitani et al., 2007; Shakhssalim et al., 2010; Chou et al., 2011; Vezzoli et al., 2011, 2015; Guha et al., 2015; Apolikhin et al., 2016; Qin et al., 2019). Some differences in the results of the investigations most likely can be explained by the specificity of the genetic characteristics of the Russian population, as well as by the peculiarities of the experimental group formation by different researchers.

An association between the rs1801197 polymorphism of the *CALCR* gene and urolithiasis was shown in the study of Qin et al. (2019). As a result of the meta-analysis (494 patients and 536 healthy individuals) performed by mentioned authors allele A of the locus rs1801197 was significantly associated with the risk of calcium urolithiasis development (OR for allele A was 1.987). According to our data, in the Russian population the OR for the A allele of the rs1801197 locus was 6.84 (*p* < 0.0001).

The relationship between the locus rs1042636 of the *CASR* gene and KSD was studied in populations of Italy, India, and Iran (Corbetta et al., 2006; Scillitani et al., 2007; Vezzoli et al., 2011).

Vezzoli et al. investigated an association between polimorphism rs1042636 (Arg990Gly) of the *CASR* gene and the risk of KSD development in Italian patients with primary hyperparathyroidism (OR for allele G (Gly) was 3.3) (Vezzoli et al., 2015).

Guha et al. showed the influence of the rs1042636 (Arg990Gly) polymorphism of the *CASR* gene at the development of urolithiasis in Indian population (OR for allele G (Gly) 2.21) (Guha et al., 2015).

The data on the role of rs1042636 (Arg990Gly) polymorphism of the *CASR* gene in urolithiasis development obtained by Shakhssalim et al. on the Iranian population are in a good agreement with the results of our study (Shakhssalim et al., 2010). In the mentioned study authors showed that patients with the AA genotype (Arg/Arg) at the rs1042636 locus showed a significantly higher serum ionized calcium compared to the patients with the Arg/Gly or Gly/Gly genotypes (OR for the Arg allele was 8.06).

The frequency of the rs1042636G allele according to dbSNP data^1^ in Europe varies from 7 to 10%, which corresponds to the data obtained in this study (the allele rs1042636G frequency in the control group in the current investigation was 12%). According to the results of our study, in Russian population the rare G allele of the locus rs1042636 may have a protective effect in relation to the KSD development. Thus, to date, in different populations different alleles of the rs1042636 locus of the *CASR* gene demonstrate an association with the risk of the urolithiasis development.

A number of studies in different countries were devoted to the investigation of the association between *ORAI1* gene polymorphisms and KSD development (Chou et al., 2011; Apolikhin et al., 2016). Thus, a study conducted in the Russian population by Apolikhin et al. revealed an association between the G allele of the *ORAI1* rs7135617 locus and an increased risk of a recurrence-free urolithiasis development (OR = 1.049). However, in the mentioned study the role of other polymorphisms of the *ORAI1* gene in the KSD was not investigated (Apolikhin et al., 2016).

In a study performed in Thai population Chou et al. studied the effect of 5 polymorphisms of the *ORAI1* gene (rs12313273, rs6486795, rs7135617, rs12320939, and rs712853) on the risk of the calcium urolithiasis development. As a result of their investigation, the higher risk of KSD development was established for the rs12313273 and rs6486795 polymorphisms carriers. For the C allele of the rs12313273 polymorphism, the odds ratio turned out to be the most significant (OR = 2.10). At the same time, the maximum risk of the nephrolithiasis development was demonstrated for the combination of C/T/C alleles at the rs12313273/rs7135617/rs6486795 polymorphic loci (OR = 2.54) (Chou et al., 2011).

In the current study all three mentioned above polymorphisms (rs12313273, rs6486795, and rs7135617) of the *ORAI1* gene were tested. Our results suggest an association of the C allele of the rs6486795 locus (OR = 2.25) with KSD development. The difference in the frequency of the alternative C allele of the rs12313273 locus between the experimental and control groups was pronounced, but did not reach a statistically significant level (25% versus 15%, χ2 = 3.125, *p* = 0.078). This is possibly due to the size limitation of the studied groups. When applying a comprehensive assessment of the cumulative effect of the rs12313273, rs6486795, and rs7135617 polymorphisms of the *ORAI1* gene on the risk of urolithiasis development, no significant data for their cumulative effect were obtained.

Thus, the data presented in the current study are suggestive for an association between the rs6486795 polymorphism of the *ORAI1* gene and the risk of calcium urolithiasis development in Russia. The results of our investigation do not contradict the data obtained by the above mentioned authors.

The analysis of the dominant and recessive inheritance models of the polymorphisms *CASR* (rs1042636), *CALCR* (rs1801197) and *ORAI1* (rs6486795) genes is important for assessing the risk of calcium urolithiasis development, and therefore it is important for the prevention of KSD. The recessive model for the *CASR* (rs1042636) and *CALCR* (rs1801197) polymorphisms, which was confirmed for this loci in the current study, allows us to predict a higher risk of urolithiasis development in patients homozygous for the risk alleles of these genes (rs1042636A in *CASR* and rs1801197A in *CALCR*).

Studying of the gene-gene interactions and investigating of the complex impact of gene polymorphisms are not less important for determining of the KSD risk development. In our study, the relationship between the loci rs1042636 of the *CASR* gene and rs1801197 of the *CALCR* gene was shown. This phenomenon requires, further, investigation.

Analysis of the association between the rs219780 polymorphism of the *CLDN14* gene and calcium urolithiasis in Russian population showed borderline *p*-value. Further, study of this association is needed to confirm the effect of rs219780 on the risk of KSD development.

## Conclusion

Thus, we showed the strong association between polymorphism rs1801197 of the *CALCR* gene and the risk of calcium urolithiasis development in Russian population. Further, investigation of the risk loci is necessary in order to assess molecular pathogenesis of calcium urolithiasis and will help to identify additional genetic factors of KSD development for better diagnostics of this complex disease.

## Data Availability Statement

The original contributions presented in the study are included in the article/Supplementary Material, further inquiries can be directed to the corresponding author.

## Ethics Statement

The studies involving human participants were reviewed and approved by the Ethics Committee of Sechenov University. Written informed consent to participate in this study was provided by the participants’ legal guardian/next of kin.

## Author Contributions

ML and TF: development of the concept and design of the study, collection of the samples, counseling of the patients, data analysis, statistics analysis, supervision, writing the text, and approval of the final version of the article. DS: collecting the samples for the study. KK, AS, AMa, and DK: molecular genetic testing and bioinformatic analysis. VK: statistics analysis and visualization. SN: participation in the article text preparation and analysis of the genetic results. AMo: review and editing. All authors contributed to the article and approved the submitted version.

## Conflict of Interest

The authors declare that the research was conducted in the absence of any commercial or financial relationships that could be construed as a potential conflict of interest.

## Footnotes

1 https://www.ncbi.nlm.nih.gov/snp/rs1042636#frequency_tab
