# Supplementary material for: Association of CASR, CALCR, and ORAI1 Genes Polymorphisms With the Calcium Urolithiasis Development in Russian Population
Source: Front Genet. 2021 May 12;12:621049. doi: 10.3389/fgene.2021.621049 (PMC8153711; doi:10.3389/fgene.2021.621049)
Supplement: Supplementary file 1 [file Table_1.DOCX]

Supplementary Table 1. Allele frequencies of 33 SNPs and their associations with risk of KSD development.

| SNP | Risk allele | Allele frequency | | Pearson`s Chi-square test | OR (95%CI) | *p*-value |
| --- | --- | --- | --- | --- | --- | --- |
|  |  | Case | Control |  |  |  |
| rs1256328 | T | 0,16 | 0,17 | 0,03629 | 0,93 (0,4406-1,963) | 0,8489 |
| rs7652589 | A | 0,41 | 0,42 | 0,0206 | 0,9596 (0,5467-1,684) | 0,8859 |
| rs6776158 | G | 0,39 | 0,38 | 0,02112 | 1,043 (0,5901-1,844) | 0,8845 |
| rs1501899 | A | 0,39 | 0,38 | 0,02112 | 1,043 (0,5901-1,844) | 0,8845 |
| rs1801725 | T | 0,15 | 0,13 | 0,1661 | 1,181 (0,5303-2,63) | 0,6836 |
| **rs1042636** | **A** | **0,96** | **0,88** | **4,348** | **3,176 (1,0469-12,0704)** | **0,03706** |
| rs1801726 | G | 0,05 | 0,11 | 2,446 | 0,4258 (0,1423-1,274) | 0,1179 |
| rs2853749 | T | 0,41 | 0,3 | 2,642 | 1,621 (0,9037-2,909) | 0,1041 |
| rs2853750 | G | 0,09 | 0,1 | 0,05816 | 0,8901 (0,3454-2,294) | 0,8094 |
| rs4754 | C | 0,24 | 0,33 | 1,987 | 0,6411 (0,3449-1,192) | 0,1586 |
| rs1126616 | T | 0,24 | 0,33 | 1,987 | 0,6411 (0,3449-1,192) | 0,1586 |
| rs12654812 | A | 0,39 | 0,39 | 0 | 1 (0,5665-1,765) | 1 |
| rs11746443 | A | 0,34 | 0,27 | 1,156 | 1,393 (0,7605-2,551) | 0,2823 |
| rs12669187 | A | 0,05 | 0,04 | 0,1163 | 1,263 (0,3291-4,848) | 0,733 |
| rs1000597 | C | 0,08 | 0,07 | 0,07207 | 1,155 (0,4025-3,316) | 0,7883 |
| rs1042138 | A | 0,1 | 0,08 | 0,2442 | 1,278 (0,4824-3,384) | 0,6212 |
| **rs1801197** | **A** | **0,94** | **0,69** | **20,73** | **6,843 (2,866-19,265)** | **3,6E-06** |
| rs4987682 | G | 0,08 | 0,08 | 0 | 1 (0,36-2,778) | 1 |
| rs4987667 | C | 0,08 | 0,08 | 0 | 1 (0,36-2,778) | 1 |
| rs4065 | C | 0,36 | 0,38 | 0,0858 | 0,9178 (0,5168-1,63) | 0,7696 |
| rs4236 | C | 0,2 | 0,19 | 0,03185 | 1,066 (0,5294-2,146) | 0,8584 |
| rs731236 | G | 0,31 | 0,3 | 0,02359 | 1,048 (0,5741-1,914) | 0,8779 |
| rs1544410 | T | 0,33 | 0,3 | 0,2086 | 1,149 (0,6325-2,088) | 0,6479 |
| rs12313273 | C | 0,25 | 0,15 | 3,125 | 1,889 (0,9273-3,848) | 0,0771 |
| rs7135617 | G | 0,42 | 0,39 | 0,1867 | 1,133 (0,6438-1,993) | 0,6656 |
| **rs6486795** | **C** | **0,3** | **0,16** | **5,534** | **2,25 (1,135-4,462)** | **0,01865** |
| rs3752472 | T | 0,02 | 0,02 | 0 | 1 (0,1381-7,242) | 1 |
| rs4142110 | C | 0,53 | 0,41 | 2,89 | 1,623 (0,9274-2,839) | 0,08911 |
| rs219781 | T | 0,19 | 0,25 | 1,049 | 0,7037 (0,3586-1,381) | 0,3057 |
| **rs219780** | **C** | **0,86** | **0,75** | **3,854** | **2,032 (0,993-4,306)** | **0,0524** |
| rs219779 | A | 0,19 | 0,25 | 1,049 | 0,7037 (0,3586-1,381) | 0,3057 |
| rs219778 | G | 0,19 | 0,25 | 1,049 | 0,7037 (0,3586-1,381) | 0,3057 |
| rs219777 | A | 0,16 | 0,26 | 3,014 | 0,5421 (0,2701-1,088) | 0,08256 |

SNPs selected for further analysis are in bold.
