# Supplementary material for: Association of CASR, CALCR, and ORAI1 Genes Polymorphisms With the Calcium Urolithiasis Development in Russian Population
Source: Front Genet. 2021 May 12;12:621049. doi: 10.3389/fgene.2021.621049 (PMC8153711; doi:10.3389/fgene.2021.621049)
Supplement: Supplementary file 2 [file Table_2.pdf]

Supplement table 2.

Primer sequences for identification of SNPs

1	mkb-1f	GTCCTCCAGGGACCCATTAGAAC
2	mkb-1r	CCCTCCACTTACCCAATAGGTAGT
3	mkb-2f	GGGGAAGGCAGGCTGTAACATTA
4	mkb-2r	CCTATTGCAAGAGCCCAATCAACA
5	mkb-3f	AGCACCCACATGTGCTGAGATAC
6	mkb-3r	CGAGATGAAGGTCACAATCTGGATC
7	mkb-4f	TCACTCATTCCTGTCTCTCCCAT
8	mkb-4r	GGGCAGGAAATAAGTGAGATCACA
9	mkb-5f	GGATGTCATCGGGTATACCGCAT
10	mkb-5r	CATAAGGAATTGTTTTTGGCTCAAAGAAC
11	mkb-6f	GCAAGTTCCAGGAAGCGAAGTGA
12	mkb-6r	CTTCTTGGATCTGTTCTTCAAGTCCT
13	mkb-7f	ATGTCTGGAACCTGAAGAGGTCA
14	mkb-7r	TCCAAATGCACGCCCACAAAAAC
15	mkb-8f	AGCTCAGACAGCCACAGCAAATA
16	mkb-8r	GCAGTAACCAAGATTTTGCCATTGT
17	mkb-9f	AGAGTGGTTGCAGATATTACCTTTATGTT
18	mkb-9r	CAACCAAGCCCTCCCAGAATTTA
19	mkb-10f	GACATTTGCATCTCAGTCCTGGAT
20	mkb-10r	GGCTGGCGACATCCCAATTTACA
21	mkb-11f	ACTGTTGATCTGTTTGTAGGTTTAGAGAG
22	mkb-11r	CATCTTCATAGGTTACAACAGTGATACCTT
23	mkb-12f	CCCAGATGCAAGCAGAAGGTCAT
24	mkb-12r	CAAAGCTCTGTGAACTGGACACTA
25	mkb-13f	AGCATTCTTCATTAGCATGGCTTTG
26	mkb-13r	CCTAATATGCAAAGGTAGTGCCAGAA
27	mkb-14f	GCAGCCTTCACAGGTCATAGCAT
28	mkb-14r	CTATGTAGGGCGAATCATGTATGAGG
29	mkb-15f	AGCCATGGTATGTACTGTGAATGC
30	mkb-15r	GGGGACAACTGGAGTGAAAACTTC
31	mkb-16f	ACACATGGAAAGCGAGGAGTTGA
32	mkb-16r	CTCAGAAGATGCACTATCTAATTCATGAGA
33	mkb-17f	GGGACCTATTGCTCACAGGAAGA
34	mkb-17r	GCTTGAAAACACTAGGAGCTGCAT
35	mkb-18f	TGTGTGCCAGCTGTGACTTAGAA
36	mkb-18r	CCCATATCCCTGAAAGCCAGGTA
37	mkb-19f	CTGTTTAAGGCCTGTCTGAGGTT
38	mkb-19r	GCTCCAGCCCACCAAGAGGATTC
39	mkb-20f	GGCCTGGGATGAGAAAGACAATA
40	mkb-20r	GTAATCGGGTAAAACGTGAGGTGAT
41	mkb-21f	CCTCACTGCAGTGCACTTTCATTA
42	mkb-21r	CCACTTTATCCTTCTCAATTTTGGCCT
43	mkb-22f	GTCTACACGAGAGTCTCACACTTC
44	mkb-22r	GAGATGCCCTGCCCTTTTTAACT
45	mkb-23f	CTTGGATACAAAAATTGCCCAGAAGT

46	mkb-23r	AGCGGCATGAAGTTTGAGATTGG
47	mkb-24f	GCCTGGCCAATCTCAAACTTCAT
48	mkb-24r	TCCTACCTGAAAGGGCTCTGGAT
49	mkb-25f	GCCCAGAGACTGGTTTTGGACAC
50	mkb-25r	GTGGCCTTATCCTGAGTTCTAGGT
51	mkb-26f	GCCGTCTCAATAATGTTTAGGACAGA
52	mkb-26r	GCTTTTAGCTCTTTGAGGAATGCTTT
53	mkb-27f	GGAAGGAAAGCTGGACTCCACTT
54	mkb-27r	CTAACACGAGGCCTAAGACCTCA
55	mkb-28f	CTCCCTCTTCTCACCTCTAACCA
56	mkb-28r	CAAAAGGAGACACAGATAAGGAAATACCT
57	mkb-29f	AATGGGCAGAACTTGGCCATAAT
58	mkb-29r	ATCAAGACAGAAGTGCTTCAAATTCTTTT
59	mkb-30f	AGTTTTCCAACATCCTGCTTTCATAGA
60	mkb-30r	ACGAGAAGAGATAAGTCACAAATGTCATT
61	mkb-31f	TCCACCACAGAACTAAGTTACTCATTAGA
62	mkb-31r	GAAGTCTAATGTCCACCCACTTTGAA
63	mkb-32f	GCGTTAGCTTCATGCTGCACTCA
64	mkb-32r	GGTATCACCGGTCAGCAGTCATA
65	mkb-33f	GGATTGGCCTTGAAAGGGAGACA
66	mkb-33r	CCCCAAACCTAGGGCTGGATTTC
